# Effects of Emotional Response on Adherence to Antihypertensive Medication and Blood Pressure Improvement

**DOI:** 10.1155/2013/358562

**Published:** 2013-02-03

**Authors:** Robert D. Keeley, Margaret Driscoll

**Affiliations:** ^1^Denver Health Medical Center, Denver, CO, USA; ^2^Department of Family Medicine, University of Colorado and Level One Physicians, Denver Health, MC 1914, 301 West 6th Avenue, Denver, CO 80204, USA; ^3^Driscoll Consulting, Boulder, CO 80303, USA

## Abstract

*Background*. Developing interventions to improve medication adherence may depend upon discovery of novel behavioral risk factors for nonadherence. *Objective*. Explore the effects of emotional response (ER) on adherence to antihypertensive medication and on systolic blood pressure (SBP) improvement. *Design*. We studied 101 adults with diabetes and hypertension. The primary outcome, 90-day “percentage of days covered” adherence score, was determined from pharmacy refill records. The secondary outcome was change in SBP over 90 days. ER was classified as positive, negative, or neutral. *Results*. Average adherence was 71.6% (SD 31.4%), and negative and positive ER were endorsed by 25% and 9% of subjects, respectively. Gender moderated the effect of positive or negative versus neutral ER on adherence (interaction *P* = 0.003); regardless of gender, negative and positive ER were associated with similarly high and low adherence, respectively, but males endorsing neutral ER had significantly higher adherence than their female counterparts (85.6% versus 57.1%, *F* value = 15.3, *P* = 0.0002). Adherence mediated ER's effect on SBP improvement: among participants with negative, but not positive or neutral, ER, increasing adherence and SBP improvement were correlated (Spearman's *r* = 0.49, *P* = 0.02). *Conclusions*. Negative, but not positive or neutral, ER predicted better medication adherence and a correlation between medication adherence and improvement in SBP.

## 1. Introduction

The World Health Organization has described nonadherence to medications as the leading cause of preventable morbidity and mortality [1]. For instance, 50% of those treated for hypertension experience clinically significant medication non-adherence, leading to worse blood pressure control, cardiovascular morbidity, higher medical costs, and increased all-cause mortality [2–5]. A recent analysis by Ho et al. found that non-adherence to treatment for hypertension was associated with higher hospitalization rates and all-cause mortality among persons with diabetes [6]. Improving adherence has been called the “next frontier in (healthcare) quality improvement” [7].

Despite decades of research, poor adherence to treatment recommendations remains prevalent across a range of disease targets, types of treatment, and populations [8]. Factors thought to be causally related to non-adherence vary broadly and include regimen complexity, poor patient-provider communication, depression, minimal social support, and financial barriers [9]. Despite this knowledge, a 2008 Cochrane review concluded that most interventions to improve medication adherence did not neither achieve sustained improvements in adherence nor lead to better clinical outcomes [10]. Steiner has recommended that improving interventions will likely depend upon better understanding of behavioral factors, as opposed to socioeconomic or clinical factors, and their relation to non-adherence [11, 12].

We previously observed and reported an association between a behavioral factor (emotional response or “ER”) and medication non-adherence (to antidepressant medication) [13]. In the current study, we ascertained baseline ER in a sample of complex patients with diabetes and comorbid hypertension. We were interested in the effect of ER on adherence to medication for hypertension and on blood pressure improvement.

Because emotions and emotional disorders are complex phenomena, their effects on outcomes may vary by factors such as gender. For instance, in a cross-sectional study of persons with diabetes, depressive symptoms exerted a substantially more negative effect on medication adherence among men than that among women [14]. Therefore, to maximize our ability to detect interactive effects, we analyzed the data with Kraemer's moderator-mediator approach [15, 16], a systematic method we have implemented previously in a national dataset [17].

## 2. Objectives

In a sample of patients with diabetes and comorbid hypertension, we conducted an analysis to explore how a measure of emotion, ER, was associated with adherence to blood pressure medication and with change in systolic blood pressure (SBP) over time. Based upon previous findings [13, 14], we hypothesized that ER (positive or negative versus neutral) would be associated with worse adherence to blood pressure medication for men than that for women.

## 3. Methods

### 3.1. Sample and Study Design

Participants were patients with diabetes and comorbid hypertension (ICD code 401.9) who were invited to join the study if they were receiving care at one of 7 participating clinics in an urban US community health care system. Subjects were excluded if they were not English speaking, were pregnant or lactating, or exhibited poor 30-day recall. Baseline data were collected between May 2008 and March 2009. The Colorado Multiple Institutional Review Board approved the study (protocol number 07-1180).

### 3.2. Measures

#### 3.2.1. Primary Outcome

*Postbaseline Adherence to Medication for Hypertension.* We obtained prospective data on pharmacy prescription refills for antihypertensive medications beginning at baseline interview and extending to 90 days. These data were used as a source of refill compliance information. Refill compliance is a broadly used, reliable and valid method to estimate adherence and has been significantly associated with other measures of compliance (self-report, pill counts), with measures of drug presence (e.g., serum drug levels), and with physiologic drug effects [18]. We selected “percentage of days covered” (PDC) as the specific method of assessing refill compliance [18, 19]. The PDC was calculated as the number of days' supply obtained during the 90-day interval divided by the number of pill-days prescribed. PDC ranges from 0% to 100%, and can exceed 100% if more than 90 days of pills are obtained for the interval.

#### 3.2.2. Secondary Outcome

*Change in SBP from Baseline to 90 Days after Baseline.* We obtained baseline and follow-up SBP values from the medical record to determine change in SBP. The follow-up “90-day” SBP values were collected from the measurement at the first clinic visit, if any, between 90 and 150 days after baseline.

### 3.3. Independent Variables

We ascertained variables referenced in previous studies as associated with postbaseline non-adherence, or having a plausible theoretical association with non-adherence*.*

*(1) Emotional Response (ER) (Variable of Primary Interest)*. Measures of emotion are linked theoretically to adherence [20], and ER has been associated with non-adherence to antidepressant medication in a prospective study [13]. We assessed ER with a grey-scale normalized, neutral* Ekman facial expression monograph* [21–24]. The Ekman monographs are reliable and valid regardless of the rater's age, gender, race/ethnicity, or cultural background. We used the standard anchor categories, “fearful,” “disgusted,” “angry,” and “sad” (negative), “surprised” and “happy” (positive), and “neutral/no emotion” [25]. We dichotomized ER (positive or negative emotional response versus neutral/no response) [13] and also examined the effects of all three levels. 

 A total of 17 other possible sociodemographic and clinical risk factors were determined from the electronic medical record and questionnaires.

*(2) Medication Beliefs.* We measured medication beliefs with *General-Overuse* and *General-Harm *scales from the “Beliefs about Medication Questionnaire,” (BMQ) [26, 27] which have been associated with medication non-adherence in complex patients [28].

*(3) Patient-Clinician Relationship.* A collaborative relationship between the patient and their clinician was measured with a 3-item scale from the Helping Alliance Questionnaire (HAQ) [29].

*(4) Self-Efficacy.* Self-efficacy [30] was measured with the *General Self-Efficacy* (GSE) scale [31–33].

*(5–10) Age, Race/Ethnicity (Non-Hispanic White, Non-Hispanic Black, Hispanic), Gender, Smoking Status (Current versus Former or Never), Insurance (Private, Medicaid or Medicare, None), and Total Medication Copay/Month.* Socio-demographic factors were assessed from the electronic medical record.

*(11–14) Social Support: Functional and Physical Assessments.* A single item inquired how mental or physical health problems affect social activities, and a 2-item bodily pain score assessed pain severity [34, 35]. Body mass index (BMI) was categorized according to National Heart Lung and Blood Institute criteria [36]. Baseline systolic blood pressure (SBP) was assessed from the medical record by averaging the 2 most recent measures.

*(15-16) Medication Complexity.* From pharmacy records, we calculated the average number of antihypertensive pills taken daily over the month prior to the initiation of the study. We also ascertained the total number of prescriptions for all medications taken for chronic conditions over the previous month.

*(17) Depressive Symptoms/Depression.* We used a valid and reliable instrument, the Patient Health Questionnaire-2 (PHQ-2, 0–6 points), to assess depressive symptoms and probable Major Depression (PHQ-2 ≥ 4, sensitivity 0.76, specificity 0.87) [37].

*(18) Prebaseline Adherence.* Prebaseline adherence to medication for hypertension over the 90 days prior to baseline was determined in a manner analogous to the measurement of postbaseline adherence.

Published reliabilities for the PHQ-2, HAQ collaboration subscale, and the BMQ scales were acceptable (Cronbach alpha coefficients > 0.70). For descriptive purposes, a coronary heart disease (CHD) risk score was generated in the electronic health record (HER) using patient age, gender, presence of diabetes, total and high density lipoprotein cholesterol, smoking status, systolic blood pressure, and whether the patient was currently taking any medication for high blood pressure [38]. This Framingham CHD risk has been reported to be moderately effective at identifying persons with diabetes who are at high risk for CHD, performing similarly in this regard to the United Kingdom Prospective Diabetes Study (UKPDS) risk engine. The UKPDS CHD risk score was not available in the study site's electronic health record [39].

### 3.4. Analytical Approach

Because we planned to explore for possible pathways to medication adherence and change in blood pressure, we chose to analyze data with Kraemer's moderator-mediator approach [40, 41].

**Step 1**

We organized the possible predictors of adherence and blood pressure change by time and domain [17]. Those possible risk factors determined prior to baseline, for example, gender which occur at conception, were flagged as potential “moderator” variables of possible predictors that were determined later (e.g., postbaseline adherence). Moderator variables define for whom or under which conditions a risk factor is clinically significant or not.

**Step 2**

We examined the univariate association between each possible predictor, and the outcomes adherence and change in SBP. Possible predictors that were significantly associated with outcome (Spearman's *r* > 0.10 and *P* < 0.20 (ordinal, dichotomous variables) or Kruskal Wallis *P* < 0.20 (categorical variables)) were selected for further analysis.

**Step 3**

In Steps 3 and 4, we examined possible predictors surviving Step 2 for interactions occurring across time. The dependent variable for these tests was either adherence or change in SBP; the two predictors of interest and their interaction were independent factors. If two risk factors occurring at different time points were not correlated (Spearman's *r* ≤ 0.10), we tested for moderators. If the interaction effect was statistically significantly different than zero, the earlier occurring predictor was considered a moderator. Correlated risk factors (Spearman's *r* > 0.10) can be assessed for mediation with the same approach (mediator occurs after the mediated variable).

**Step 4**

Because predictors for an outcome often vary within moderator subgroups, if a moderator variable was noted, the algorithm was repeated within each subgroup defined by the moderator.

*Statistical Significance.* To limit probability of Type I error in the moderator-mediator analyses, we set the two-tailed alpha at *P* < 0.025. For other analyses, for example, a simple correlation between adherence and change in SBP, we set the significance level at *P* < 0.05. For analyses within moderator subgroups we conducted backward regression, entering variables associated with adherence at Spearman's *r* > 0.10 and *P* < 0.20, and removing variables for *P* > 0.20. For multivariate analyses, we used SAS 9.2 and PROC SURVEYREG nested by clinic site with variables centered [42].

## 4. Results

### 4.1. Characteristics of the Participants

We recruited 101 subjects, 54 at the time of their visit with their primary care clinician and 47 by telephone invitation. There were no differences in mean clinical parameters (hemoglobin A1C, SBP) between patients recruited at the time of the visit or by telephone. 

The mean age of the participants was 52.2 years, and 39% were non-Hispanic White, 27% were non-Hispanic Black, and 34% were Hispanic. Fifty-one (50.5%) had no insurance, and 49.5% were on Medicaid, Medicare, or private insurance. The mean depressive symptom score was 2.0 (SD 1.7, range 0–6), and 15.8% had probable major depression. Among patients age 30 or higher without a documented history of cardiovascular disease in the electronic health record, average Framingham coronary heart disease risk was 13.4%. Eighteen subjects (17.8%) had established cardiovascular disease.

Patients were prescribed a total of 6.4 (SD 2.8, range 2–16) prescriptions for medications, and averaged 1.9 (SD 1.9 range 1–6) medications for blood pressure. Ninety (90.1%) subjects were taking an angiotensin-converting enzyme inhibitor or angiotensin receptor blocker medication, 41 (40.6%) were taking a blood pressure medication with beta-blocking activity, and 17 (16.8%) were taking a calcium channel-blocking antihypertensive medication. Four participants did not fill any prescriptions for antihypertensive medication during the study and received a 0% adherence score. Four different subjects were not taking medication for diabetes (insulin or oral antihyperglycemic medication) and were presumably diet controlled as only one of these 4 had a hemoglobin A1C over 8.0%, demonstrating suboptimal control.

A substantial proportion of the sample, 37.6%, were current smokers. The average body mass index was relatively high at 34.0, demonstrating obesity.

The mean baseline SBP and hemoglobin A1C were 135.9 mm Hg and 9.0%, respectively. At baseline, men and women were taking an identical number of blood pressure pills daily (1.9 versus 1.9, *F* value = 0.0001, *P* = 0.99).

Similar to previous findings [13], about one-third (34%) of subjects reported an emotional response (*n* = 1 with missing ER). One-fourth endorsed a negative ER, 9% a positive response, and 66% a neutral response. ER was not associated with gender, race/ethnicity, or depression.

The mean postbaseline percent, of days covered (PDC) adherence score was 71.6% (SD 31.4, range 0%–130%). 

### 4.2. Moderator-Mediator Analysis of Medication Adherence (Table 1(A), Figure 1)

Univariate factors associated with adherence to blood pressure medications included (1) gender; (2) race/ethnicity; (3) depressive symptoms; (4) probable depression; (5) ER; (6) prebaseline adherence.

Gender was not correlated with depressive symptoms, depression, or ER (*R* values < 0.1, *P* values = NS). Gender did not interact significantly with depression or with depressive symptoms. Gender moderated the effect of ER on adherence (interaction *P* = 0.003). The moderating effect remained significant (*P* = 0.016) when pre-baseline adherence was omitted from the model. 

### 4.3. Neutral ER and Prospective Adherence

Males and females with any ER had similar PDC adherence scores, 70.1% (SD 35.1) and 72.4% (SD 34.1), respectively. The differential effect of gender on prospective adherence was specific to those participants endorsing neutral ER. Notably, males endorsing neutral ER were adherent (PDC = 85.6%, SD 24.1, *n* = 34), while their female counterparts were less adherent (PDC = 57.1%, SD 34.1%, *n* = 32), a highly significant difference (*F* value = 15.3, *P* = 0.0002). Because gender moderated the effect of ER on adherence, we repeated the analytical approach for males and females separately.

### 4.4. Adherence among Males (Table 1(B))

Among males, positive ER (*n* = 7) was associated with a mean adherence score of 50.8%, while neutral (*n* = 34) and negative (*n* = 10) ER were associated with 85.6% and 83.6% adherence, respectively. We dichotomized the variable as positive ER versus neutral or negative ER for further analysis.

Univariate correlates of adherence included (1) total number of prescriptions received/month; (2) depressive symptoms; (3) probable depression; (4) positive ER; (5) pre-baseline adherence.

 In the final model, pre-baseline adherence (*F* value = 28.1, *P* < 0.0001) was associated with better prospective adherence, and positive ER (*F* value = 12.3, *P* = 0.001) and depressive symptoms (*F* value = 5.1, *P* = 0.03) were associated with worse adherence. The 7 males with a positive response included in the analysis had a lower average adherence than their 44 counterparts with a neutral or negative response, 50.8% (SD 40.3) versus 85.1% (SD 23.4), respectively (Cohen's *d* = −1.04, large ES). No mediator variables were uncovered.

### 4.5. Adherence among Females (Table 1(B))

Among females, negative ER (*n* = 15) was associated with a mean PDC of 74.3%, while neutral (*n* = 32) and positive (*n* = 2) ER were associated with 57.1% and 57.8% PDC, respectively. We dichotomized ER as negative versus neutral or positive ER for further analysis. 

 Univariate correlates associated with adherence included: (1) age; (2) total medication copay/month; (3) negative ER; (4) any insurance; (5) bodily pain; (6) pre-baseline adherence.

 Independent predictors of better adherence in the final model included pre-baseline adherence (*F* value = 14.4, *P* = 0.0005), negative ER (*F* value = 6.3, *P* = 0.02), and age (*F* value = 3.4, *P* = 0.07). Females with negative ER averaged a 74.3% (SD 24.3) adherence, while their counterparts with neutral or positive ER averaged 57.1% (SD 33.6) adherence (*d* = 0.58, medium ES). No mediator variables were uncovered.

The effects of ER on postbaseline adherence remained significant when pre-baseline adherence was omitted from the models for both males and females.

### 4.6. Moderator-Mediator Analysis of Change in Systolic Blood Pressure (Table 1(C))

The mean improvement in SBP across the population was 5.9 mm Hg (SD 17.8) (*n* = 95). Males and females endorsing neutral ER realized average improvements in SBP of 2.5 (SD 16.5) and 4.7 (SD 16.2) mm Hg, respectively. Relative to subjects endorsing negative or neutral ER, those endorsing positive ER had the worst adherence rate of 52.3% (SD 39.3), yet paradoxically appeared to experience the most improvement in SBP at 14.8 (SD 15.8) mm Hg. For subjects endorsing negative ER, the improvement in SBP averaged 10 mm Hg.

Univariate correlates associated with change in SBP included (1) baseline SBP; (2) ER (3 levels); (3) depressive symptoms; (4) self-efficacy; (5) non-Hispanic White race/ethnicity; (6) number of blood pressure pills taken per day.

**Figure 2:**
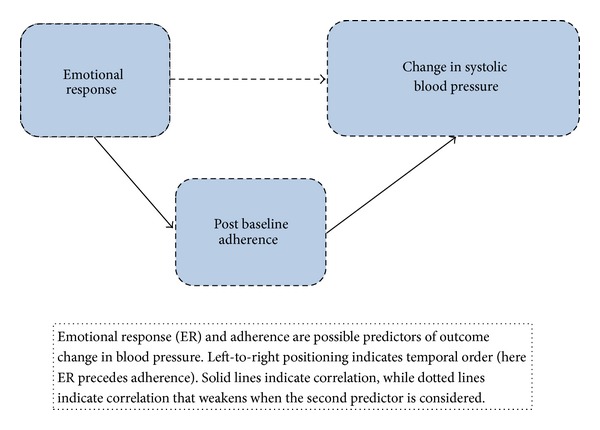
Mediator model.

There were no moderator variables, and there was no association between ER and adherence across the complete sample (*r* = −0.06, *P* = 0.54). Because negative ER was associated with excellent adherence and blood pressure improvement, we tested whether prospective adherence mediated the effect of negative ER on improvement in SBP. In other words, we examined whether adherence might explain why ER was significantly associated with change in SBP for at least a subset of subjects, those with baseline negative ER. In an adjusted model, adherence (*P* = 0.003) and the interaction between negative ER and adherence (*P* = 0.018) predicted improvement in SBP, while negative ER (*P* = 0.048) was not significant. The results suggest that adherence mediated or explained some of the effect of ER on change in SBP (Figure 2). 

 For subjects endorsing negative ER, better adherence was closely associated with improvement in SBP (*r* = 0.49, *P* = 0.02). Associations between adherence and change in SBP were small and not significant for groups of subjects endorsing positive ER (*r* = −0.18, *P* = 0.7) or neutral ER (*r* = 0.04, *P* = 0.8). Adherence explained the effect of ER on change in SBP for subjects with negative ER, but not for subjects with neutral or positive ER, demonstrating that adherence appeared to partially mediate the effect of ER on blood pressure improvement.

## 5. Discussion

Non-adherence to medications for hypertension and other chronic health conditions is a public health problem, and most efforts to improve adherence have not led to sustained improvements in adherence or in clinical outcome [1]. Improving outcomes for complex patients with hypertension will likely depend upon uncovering and clarifying the effects of moderators and mediators that comprise pathways to adherence and blood pressure improvement [16]. In this study, we provide initial descriptions of two factors, gender and ER, that defined pathways to adherence. Three factors, gender, ER, and adherence, defined pathways to blood pressure improvement. Gender appeared to moderate the effect of ER on adherence, and adherence appeared to mediate the effect of ER on blood pressure improvement, at least for patients with baseline negative ER. This is the first study of which we are aware to use a moderator-mediator analysis in order to begin mapping pathways to blood pressure medication adherence, and from adherence to change in blood pressure.

Theoretically, predictors for an outcome should vary within subgroups defined by a moderator. Although the relationship between ER and adherence was somewhat more complex than hypothesized, we found that the predictors of worse adherence for males—depression and positive ER—were different than the primary predictor among females—neutral ER. The results suggest that development of more effective interventions to improve adherence to antihypertensive medication might need to be tailored according to gender. 

We learned that for male and female subjects endorsing negative ER, but not neutral or positive, ER, adherence and improvement in blood pressure were tightly correlated. With the exception of males with neutral ER, adherence was low for participants with neutral or positive ER. Paradoxically, these two groups of subjects were characterized by a lack of correlation between adherence score and change in SBP, yet essentially normalized their SBP over the study period. Pathways to blood pressure improvement among complex patients with hypertension endorsing neutral or positive ER may involve other factors, for example, change in diet or weight, that we did not measure. Such patients may be more likely to take their blood pressure medications just prior to a visit with their doctor, thus appearing to normalize their blood pressure despite experiencing overall poor adherence.

Similar to findings from Nau et al. [14], we found that depressed males but not females experienced diminished adherence relative to their nondepressed counterparts. However, depression is not necessarily causally related to medication non-adherence: in one study, improving depressive symptoms among persons with hypertension did not lead to improved adherence to medication for high blood pressure or to improved blood pressure improvement [43]. 

The associations between negative ER, high adherence to antihypertensive medication, and improvement in blood pressure may be explained in several ways. On the one hand, patients with hypertension may directly endorse negative ER as a physiological consequence of elevated blood pressure, that is, elevated blood pressure may shift some persons into a state of negative ER, which then drives corrective behaviors such as improved medication adherence to address the threat to health. On the other hand, negative ER may represent a trait that is consistent over time and is typified by vigilant attention to environmental cues such as recommendations to take medication [20].

Neutral ER may serve as a maintenance factor steering the individual to follow a default behavior pattern. Lower adherence for females and higher adherence for males endorsing neutral ER was associated with somewhat elevated baseline SBP. However, both males and females endorsing neutral ER experienced similar 2–5 mm improvements in SBP over 90 days, leading to a normalized average SBP of 130 mm Hg for persons with diabetes. Low adherence in the 57% range for females endorsing neutral ER may not in itself be maladaptive, as SBP was normalized over time.

Positive ER appears to be relatively uncommon, occurring in about 10% of primary care patients in both this and one previous study [13]. Because of its low prevalence, future studies of the effects of positive ER on adherence will require substantially expanded recruitment.

We note several limitations to our study findings. Threats to external validity include methodological limitations. (1) Patients were recruited with 2 approaches—by phone or prior to a clinical visit. (2) This is a modest and highly heterogeneous convenience sample with patients of different race-ethnicities from one health system. However, despite these limitations we were able to demonstrate significant effects of ER on adherence. The sample's overall glucose control was relatively poor at 9.0%, while baseline average systolic blood pressure was more modestly elevated at 135.9 mm HG. While it is unclear why patients with diabetes in the sample appeared to have better blood pressure than diabetes control, it should be noted that the relative difference extended to all persons with diabetes attending the health care system, where the average SBP was 135.0 mm Hg and average A1C was 8.5% in 2008. 

It is possible that confounders might better explain the associations between ER, adherence, and blood pressure improvement that we describe, and only larger studies powered for exclusive stratification and subgroup analyses will allow researchers to fully address this potential issue. It is likely that Kraemer's emphasis on interaction tests allowed the research team to avoid some potential pitfalls associated with extensive stratification, including “limited extent of data available …and premature claims of subgroup findings” [44].

Threats to internal validity include the use of a brief measure of probable major depression, the PHQ-2. However, the PHQ-2 has been shown to perform similarly to the full PHQ-9 to detect depression in primary care [45]. While the theoretical underpinning of the role of emotion in human behavior is extensively researched, the use of a specific measure, emotional response to neutral facial expression, is relatively novel. However, the Ekman monographs used to ascertain emotional response are highly valid and reliable tools, and prospective associations with adherence to blood pressure medication and with blood pressure improvement demonstrate additional face and predictive validity. It would be important to conduct comprehensive reliability and validity testing of the ER construct. Because no gold standard for ascertaining medication adherence exists, future studies would include multiple measures.

This is the first study of which we are aware to investigate how ER may be associated with medication adherence and clinical outcome. It is also the first study to demonstrate that negative ER may positively influence both adherence to antihypertensive medication and subsequent improvement in SBP among complex primary care patients. The results of this investigation should be considered hypothesis generating, and future studies would further validate ER and evaluate these initial descriptions of effects of ER on adherence to blood pressure medication and on blood pressure improvement. 

## Figures and Tables

**Figure 1:**
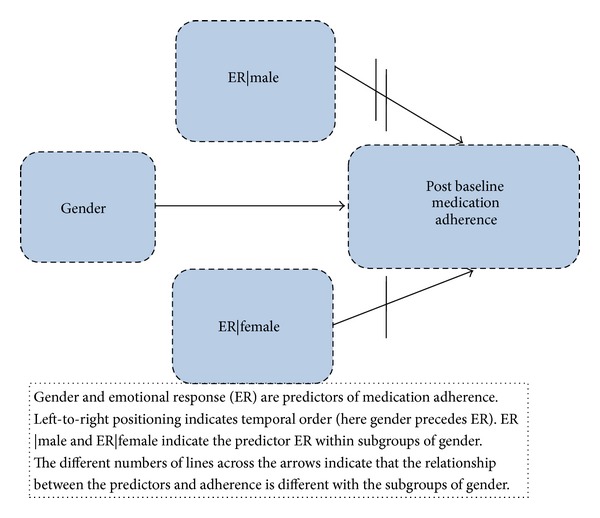
Moderator model.

**Table 1:** Multivariate models.

	*F*-value	Pr(>|*t*|)
(A) Moderator model (*n* = 89*)		
Outcome = postbaseline 90-day adherence		
Model	23.5	<0.0001
Intercept	4.8	0.03
Prebaseline adherence	41.5	<0.0001
Male	2.7	0.10
ER	0.00	0.98
Gender × ER	9.7	0.003
(B) Models stratified by gender		
Outcome = postbaseline 90-day adherence		
*Males* (*n* = 44^†^)		
Model	28.6	<0.0001
Intercept	3.7	0.06
Prebaseline adherence	28.1	<0.0001
Positive ER	12.3	0.001
Depressive symptoms	5.1	0.03
*Females* (*n* = 41^#^)		
Model	5.6	<0.0001
Intercept	5.9	0.02
Prebaseline adherence	14.4	0.0005
Age	3.4	0.07
Negative ER	6.3	0.02
(C) Change in blood pressure model (*n* = 94^‡^)		
Outcome = change in SBP		
Model	5.6	0.0005
Intercept	14.8	0.0002
Baseline SBP	12.6	0.001
Negative ER	4.0	0.048
Postbaseline 90-day adherence	9.3	0.003
Negative ER × adherence	5.9	0.016

*n = 1 with missing emotional response data, *n* = 11 with no prebaseline adherence data. *R*-square = 0.46; ^†^ *n* = 7 with missing pre-baseline adherence data, *R*-square = 0.64; ^#^ *n* = 9 with missing independent-variable data. *R*-square = 0.42; ^‡^ *n* = 1 with missing independent-variable data and, *n* = 6 with missing dependent-variable data. *R*-square = 0.24. ER: emotional response; SBP: systolic blood pressure.
